# Diagnosis and Treatment of Alcohol-Dependent Patients With Comorbid Psychiatric Disorders

**Published:** 1999

**Authors:** Vania Modesto-Lowe, Henry R. Kranzler

**Affiliations:** Vania Modesto-Lowe, M.D., M.P.H., is an assistant professor of psychiatry and Henry R. Kranzler, M.D., is a professor of psychiatry in the Department of Psychiatry at the University of Connecticut School of Medicine, Farmington, Connecticut

**Keywords:** diagnosis, treatment, AODD (alcohol and other drug dependence), behavioral and mental disorder, dual diagnosis, comorbidity, epidemiology, drug therapy, psychotherapy, literature review

## Abstract

Psychiatric disorders occur more often among alcoholics than among the general population. The psychiatric disorders that alcoholics most frequently experience include mood disorders (e.g., depression), anxiety disorders, and antisocial personality disorder. The evaluation of psychiatric symptoms in alcoholic patients is complicated by the multiple relationships that exist among heavy drinking, psychiatric symptoms, and personality factors. For example, alcoholics with co-occurring depression may be at greater risk of psychosocial problems, relapse, and suicide. Conversely, heavy drinking may produce or worsen symptoms of depression or anxiety. Although clinical experience provides general guidance for treating these patients, further research is needed to develop effective psychosocial and pharmacological therapies aimed at specific combinations of psychiatric and addictive disorders.

Epidemiologic and clinical studies suggest that an alcoholic involved in treatment is statistically at high risk for a psychiatric disorder compared with the general population. Conversely, certain psychiatric disorders are often accompanied by alcohol abuse or alcohol dependence (Meyer 1986). The psychiatric disorders that occur most frequently and that have been studied most in alcoholics are mood disorders (e.g., depression), anxiety disorders, and antisocial personality disorder (ASPD).

The presence of comorbid psychiatric disorders in alcoholic patients has clinical and prognostic implications. For instance, alcoholics with comorbid depression may be at greater risk of psychosocial and interpersonal problems, treatment noncompliance, alcoholic relapse, and attempted and completed suicide (Kranzler et al. 1998). Additionally, heavy drinking may produce or worsen depressive or anxiety symptoms. Research also indicates that alcoholics with ASPD start drinking at an earlier age and develop symptoms of dependence sooner than alcoholics without ASPD (Hesselbrock et al. 1992).

Together, these findings suggest that patients diagnosed with an alcohol-use disorder should undergo thorough psychiatric assessment. Conversely, patients seen in psychiatric settings should be routinely evaluated for the presence of an alcohol-use disorder. However, the evaluation of psychiatric symptoms in alcoholic patients can present a challenge to the clinician because of the complexity of the relationships between heavy drinking, psychiatric symptoms, and personality factors.

This article explores the extent of comorbidity between psychiatric disorders and alcohol-use disorders, describes research on the nature of comorbid relationships, and suggests general treatment considerations as well as treatment strategies aimed at specific comorbidities. The article also considers pharmacological and psychosocial approaches, both separately and in combination.

## Epidemiology

Of the various epidemiologic studies that have examined comorbidity among alcoholics sampled from the general population, the National Comorbidity Survey (NCS) provides a representative sample and covers psychiatric disorders comprehensively (Kessler et al. 1994). This community survey of more than 8,000 respondents showed that among women, 72.4 percent of those who abused alcohol and 86.0 percent of those who were alcohol dependent had a comorbid lifetime psychiatric or drug-use disorder (Kessler et al. 1997). Among men, the comparable figures for alcohol abuse and alcohol dependence were 56.8 percent and 78.3 percent, respectively (Kessler et al. 1997). The NCS also shows that anxiety and mood disorder were the most common comorbid disorders among female alcoholics, whereas drug-use disorders and ASPD were the most common comorbid psychiatric disorders among male alcoholics (Kessler et al. 1997). Despite some gender differences, NCS shows that for both sexes, the association between alcohol dependence and anxiety disorders, mood disorders, and ASPD was significantly elevated over what might be expected by chance.

High rates of comorbid lifetime psychiatric disorders also have been observed in alcoholic patients in clinical settings. Powell and colleagues (1982) found that 63 percent of 565 male inpatient alcoholic veterans met lifetime criteria for a comorbid psychiatric diagnosis. The most common comorbid diagnosis was depression (42 percent), followed by mania (20 percent) and ASPD (20 percent). Similarly, in a study of 321 inpatient alcoholics, Hesselbrock and colleagues (1985) found that 75 percent of men and 80 percent of women received one or more lifetime comorbid diagnoses. Drug abuse was the most prevalent lifetime diagnosis (45 percent), followed by ASPD (41 percent), major depression (38 percent), phobia (27 percent), obsessive-compulsive disorder (12 percent), panic disorder (10 percent), mania (4 percent), and schizophrenia (2 percent).

## The Relationship Between Alcoholism and Psychiatric Disorders

Alcoholics often report that they drink to relieve a dysphoric mood, which has been termed “self-medication.” This hypothesis embodies a view of alcoholism in which psychiatric symptoms are primary, with drinking occurring in response to those symptoms. It has been shown, however, that both chronic heavy drinking and alcohol withdrawal can exacerbate negative mood states. This intensification of symptoms may result from the pharmacological effects of alcohol or from psychosocial problems (e.g., family, work, or legal problems) that can stem from chronic heavy drinking (Kranzler et al. 1998). Because depressive symptoms have been shown to be both a cause and a consequence of heavy drinking, a careful history is required to guide treatment decisions.

Patients with bipolar disorder may drink to alleviate both manic and depressive symptoms, though evidence indicates that the greatest risk for heavy drinking occurs during the manic phase of their illness (Kranzler et al. 1998). Conversely, alcohol intoxication in persons without bipolar disorder may produce symptoms similar to those associated with hypomania or mania. Such symptoms may include elevated mood, grandiosity, irritability, or physical agitation. Some of these symptoms are also commonly found during alcohol withdrawal, which further complicates the diagnosis of bipolar disorder in alcoholics.

The relationship between drinking and anxiety disorders is also complicated. Alcoholics often report intense, but transient, alcohol-induced anxiety symptoms (i.e., palpitations, sweatiness, lightheadedness, and fear of objects or social situations), which often abate with abstinence from alcohol. On the other hand, individuals with agoraphobia or social phobia may drink to self-medicate anxiety symptoms; those with panic disorder and generalized anxiety disorder may experience these symptoms as a consequence of heavy drinking (Kranzler 1996).

Significant overlap also exists for symptoms of alcohol dependence and personality disorders. This finding is particularly true for ASPD, because alcoholics often exhibit antisocial behavior associated with their heavy drinking (e.g., lying, impulsive aggression, and breaking the law). Clinicians should work with patients to differentiate between symptoms that are attributable to alcohol dependence and symptoms that are attributable to ASPD. However, this task may be difficult, because although patients may be able to distinguish whether anxiety symptoms are related to substance intoxication or withdrawal, they may have greater difficulty differentiating between antisocial behaviors that are alcohol-related (e.g., disorderly conduct caused from intoxication) from those that are not (e.g., chronic lying).

## Treatment of Patients With Comorbid Alcoholism and Psychiatric Disorders: General Considerations

Initial treatment for alcohol dependence consists primarily of detoxification (i.e., treatment of acute physical withdrawal symptoms) and psychosocial strategies aimed at maintaining abstinence. The added presence of a comorbid psychiatric disorder can substantially complicate this approach. For instance, depressive symptoms (e.g., decreased energy and interest) can interfere with a person’s attendance at Alcoholics Anonymous (AA) meetings or group psychotherapy sessions, two widely used psychosocial interventions for alcoholism. Similarly, patients with anxiety symptoms (e.g., panic attacks, social phobia) may find it difficult to attend AA meetings or to learn skills for coping with situations that represent a high risk for alcoholic relapse. Alcoholics with ASPD, on the other hand, may show manipulative or aggressive behaviors, which can cause significant interpersonal problems for themselves and for others in treatment.

The diversity of alcoholics with comorbid disorders means that individualized treatment approaches may more effectively address the comorbidity. However, with the exception of studies of antidepressant therapy for depressed alcoholics, little research exists on how best to treat alcoholics with comorbid psychiatric disorders. Although cognitive behavioral therapy (CBT) has been shown to be useful in treating patients with alcohol dependence, as well as those with certain depressive and anxiety disorders, limited research exists on the utility of integrated psychotherapeutic interventions that address the particular needs of alcoholics with comorbid disorders.

Despite the lack of systematic study, some basic principles may be helpful in approaching patients with comorbid alcohol use and mood or anxiety disorders. The clinician’s first objective is to establish both an alliance with the patient and a common goal for treatment. The goal in treating a patient with major depression is to reduce the depressive symptoms and return the individual to a normal level of function. Similarly, in treating a patient with alcohol dependence, the goal is usually abstinence, in order to restore normal function.

Although it may be obvious that the appropriate treatment goal for patients with dual diagnosis is to address both the substance use and the psychiatric symptoms, this simple fact is often overlooked. Thus, it is important to emphasize that treatment of such patients requires dual goals: namely, abstinence from alcohol and stabilization of comorbid symptoms, both of which can be expected (and may be required) to improve the person’s health and psychosocial functioning.

Insofar as these goals can be achieved using psychosocial or pharmacological treatments, or a combination of the two, clinicians must consider the optimal sequence of interventions. Efforts to enhance motivation for recovery can be initiated during the first contact with the patient, which for some patients may be during detoxification. This can be accomplished by providing nonjudgmental feedback to the patient concerning the specific medical, social, interpersonal, or psychiatric effects of that person’s drinking on his or her life. Relapse prevention strategies can be added after detoxification is complete, assuming that the patient is adequately motivated for such treatment.

The focus of CBT is on the acquisition of skills, which may be used to manage high-risk drinking situations, or to reduce anxiety or depressive symptoms. Consequently, it makes conceptual sense to mix and match CBT techniques that are used both for relapse prevention and to treat anxiety or depressive disorders. The varying combinations make it possible to tailor a program according to the specific needs of the patient. For instance, since dysphoria is often a cue for drinking, teaching patients to avoid high-risk drinking situations can go hand in hand with teaching patients how to manage their depressed moods.

Because many psychiatric symptoms subside with abstinence, the use of medications to alleviate such symptoms should generally be postponed until at least 1 or 2 weeks of abstinence have been achieved. However, under certain clinical circumstances (e.g., severe symptoms and a clear history of a primary psychiatric disorder that was medication responsive), more immediate action may be required. In other cases, the assessment of symptoms at regular intervals throughout treatment will help to determine whether medications are indicated. Meanwhile, the patient can begin to learn skills to handle high-risk situations and craving along with techniques for managing anxiety or depression, as indicated.

Although alcoholics with comorbid disorders may find AA useful, these alcoholics often require extra encouragement to initiate and continue to attend fellowship meetings. For example, a person may need to attend several AA groups before choosing the one with which he or she feels most comfortable. Dual-diagnosis patients also may have difficulty relating to other AA members whose lives may improve more rapidly than theirs as a consequence of abstinence from alcohol. Patient education, an important aspect of treatment, should include a discussion of how psychiatric symptoms and drinking may affect one another. For instance, chronic heavy drinking may produce depressive symptoms. On the other hand, untreated depression can precipitate relapse, which in turn can augment feelings of worthlessness, hopelessness, and guilt.

Frequently, recovering alcoholics believe that recovery requires a medication-free state. Although this view is not a formal position of AA, some members hold this view. Unfortunately, this belief may reduce medication compliance in alcoholic patients with comorbid psychiatric disorders. Therefore, the prescribing physician should discuss the possible benefits and adverse effects of prescribed medications to treat psychiatric symptoms and any potential interactions they may have with alcohol.

After treatment is initiated, the clinician must monitor both drinking behavior and psychiatric symptoms, because alcohol dependence, anxiety, and depressive disorders all tend to have a relapsing course. Objective methods to assess drinking include the use of breath-alcohol testing as well as measurement of liver enzyme levels, such as gamma-glutamyl transpeptidase (GGTP). GGTP, although nonspecific, is often elevated in heavy drinkers, making it of potential value as an indicator of drinking status. With comorbid depression or anxiety disorders, symptoms also should also be assessed on a regular basis, preferably with a semi-structured interview, such as the Hamilton Anxiety Rating Scale (Hamilton 1959), or a self-report questionnaire, such as the Beck Depression Inventory (Beck et al. 1961). Particular attention should be given to the risk for suicide, as it may be particularly high in depressed alcoholics (Kranzler et al. 1998). In addition, any pharmacotherapy targeting either alcohol dependence or a comorbid psychiatric disorder should be monitored regularly. In so doing, it is important to consider how biochemical changes in the liver and medical disorders associated with long-term alcohol misuse may influence medication effects.

In summary, interventions aimed at alcoholics with comorbid psychiatric disorders should address both the alcohol dependence and the comorbid psychiatric disorder. Specifically, an effort should be made to assess and enhance motivation, engage and retain patients in treatment, and educate the patient on the relationship between alcohol use and psychiatric symptoms. The judicious use of medications for persistent anxiety or depressive states can augment the psychosocial and educational efforts. Interventions found to be useful in the treatment of alcoholics with comorbid mood and anxiety disorders are discussed in the following section. A framework is also provided for the treatment of alcoholics with comorbid ASPD.

### Comorbid Alcohol Dependence and Mood Disorders

To date, studies of the pharmacological treatment of depression in alcoholics have examined tricyclic antidepressants (TCAs) and selective serotonin reuptake inhibitors (SSRIs). These studies have focused on whether antidepressants are effective in reducing depressive symptoms in alcoholics and whether treating depression in these patients has a beneficial effect on drinking behavior. Although early studies showed TCAs to be no better than placebo for the treatment of depression in alcoholics, this lack of efficacy may have been attributable to the methodological shortcomings of these trials (Ciraulo and Jaffe 1981). For example, the dosage of TCAs used in these studies was probably inadequate to produce a therapeutic effect. Recent studies of TCAs in depressed alcoholics have shown both impramine and desipramine to be effective antidepressants in alcoholics (Kranzler et al. 1998). These studies, however, do not provide consistent evidence that the effective treatment of depression has beneficial effects on drinking outcomes. Furthermore, despite the availability of low-cost generic forms of these medications, their use in depressed alcoholics is problematic given the potential for a lethal overdose associated with TCAs.

Although not as well studied in depressed alcoholics, SSRIs and other serotonergic antidepressants have a good safety profile. One study of severely depressed alcoholics showed that fluoxetine is efficacious in reducing both depressive symptoms and drinking behavior (particularly heavy drinking) in depressed alcoholics (Cornelius et al. 1997). Consistent with this is a study of sertraline (another SSRI), which showed that this agent decreases depressive symptoms in depressed alcoholics (Roy 1998). That study was deemed by the author to be of insufficient duration to examine effects on drinking behavior. More recently, a study with the non-SSRI antidepressant medication nefazodone (Serzone^®^) showed it to be beneficial in decreasing depressive symptoms in depressed alcoholics (Roy-Byrne et al. in press). However, nefazodone showed no effect on drinking behavior. Results from other studies of serotonergic antidepressants should soon be published.

The psychotherapeutic treatment of comorbid alcohol and depressive disorders has received little research attention. One randomized trial in alcoholics with high levels of depression conducted in a substance abuse partial-hospitalization setting (Brown et al. 1997) compared the efficacy of the cognitive-behavioral therapy of depression (CBT–D) to a control relaxation group. This study showed that the CBT–D group had significantly greater reductions in depression and anxious mood during the study than did the control group. Furthermore, at 6-month followup, patients in the CBT–D group had significant reductions on drinking measures and attended AA meetings more frequently than did patients in the control group.

To date, no controlled studies of either a specific psychotherapeutic approach or a pharmacological treatment for bipolar disorder in alcoholics has been published. Although both lithium and the anticonvulsants valproate and carbamazepine have demonstrated efficacy in the treatment of mania, limited evidence suggests that substance abusers tend to have subtypes of bipolar disorder (i.e., mixed and rapid cycling) that are less responsive to lithium (Donovan and Nunes 1998). This possibility, coupled with the potential toxicity of lithium in overdose, has led clinicians to preferentially employ anticonvulsants in the treatment of bipolar alcoholics.

### Comorbid Alcohol Dependence and Anxiety Disorders

Benzodiazepines, sometimes used to treat anxiety in alcoholics, are themselves subject to abuse. Therefore, a number of studies of buspirone, a nonbenzodiazepine anti-anxiety medication, have been conducted in this patient group. Of four such published studies, three showed buspirone to significantly enhance treatment retention (Kranzler 1996). These three studies also showed other beneficial effects of buspirone over a placebo, including significantly greater reduction in anxiety. In one study (Kranzler 1996), reductions in anxiety among patients with the highest levels of anxiety were associated with reduced frequency of drinking.

The two main advantages of using buspirone in alcoholics with persistent anxiety are the absence of both addiction potential and additive effects (with alcohol) on brain function, including eye-hand coordination. However, one disadvantage of buspirone is that it takes at least 2 weeks of treatment at a therapeutic dosage to exert its anti-anxiety effects. As alcoholic patients often are poorly tolerant of delayed relief of symptoms (in contrast to the rapid relief that may be provided by alcohol or treatment with a benzodiazepine), they may not accept this medication. Consequently, it is critical that buspirone treatment be combined with patient education to promote realistic expectations of the time course of effects of the medication and concomitant psychosocial treatment to sustain the patient’s commitment to treatment. Furthermore, it may be necessary to prescribe buspirone at the highest recommended dosage (i.e., 60 milligrams per day [mg/day]) to obtain a good treatment response.

Despite evidence that a variety of antidepressants, including TCAs, monoamine oxidase inhibitors (MAOIs), and SSRIs, are efficacious in the treatment of panic disorder, there are no published controlled trials of the pharmacological treatment of panic disorder in alcoholics. The use of both TCAs and MAOIs in alcoholics requires careful consideration. As mentioned, TCAs can be lethal in overdose, which is related to their capacity to produce cardiac arrthymias. Similarly, the use of MAOIs in alcoholic patients is contraindicated because of the restrictions placed on certain foods, medications, and alcoholic beverages to ensure the safe use of these medications.

Although the efficacy of the SSRIs (e.g., fluoxetine, sertraline, paroxetine) has not been evaluated in alcoholics with panic disorder, these agents are the safest. However, abstinent alcoholics may be at increased risk for experiencing jitteriness and irritability with SSRI use. Thus, an effort should be made to minimize these side effects by starting with a low dosage and gradually increasing it to a therapeutic dosage. Finally, the use of benzodiazepines (e.g., diazepam, alprazolam) beyond detoxification in alcoholics with panic disorder is probably ill advised, given their addiction potential and additive CNS depressant effects when taken in combination with alcohol.

Limited research also exists on psychotherapeutic interventions for alcoholics with anxiety disorders. One of the few studies to address this issue (Ormrod and Budd 1991) showed that among 36 anxious alcoholics, those receiving anxiety management and relaxation training experienced greater decreases in anxiety levels than did a control group that received “health education.” However, no effect was observed on drinking outcomes, either during treatment or during a 3-month followup period. Together with findings described previously for buspirone, it seems that the combination of psychotherapy and pharmacotherapy may have uniquely beneficial effects in anxious alcoholics. A carefully controlled trial of the combined approach is necessary.

### Comorbid Alcoholism and Antisocial Personality Disorder

Clinicians faced with treating patients with comorbid alcoholism and ASPD may find the task a frustrating one. However, the widespread view that alcoholics with ASPD respond poorly to treatment may be invalid, particularly in light of studies showing that some substance abusers with ASPD (e.g., those with a concomitant diagnosis of depression**)** do benefit from treatment (Alterman and Cacciola 1991; Cacciola et al. 1995). One study, for instance, showed no difference in treatment response among alcohol- and cocaine-dependent patients when groups based on the presence or absence of comorbid ASPD were compared (Cacciola et al. 1995). Similarly, a study by Longabaugh and colleagues (1994) showed that employed ASPD alcohol abusers responded as well to abstinence-focused cognitive-behavioral therapy as did a heterogeneous group of non-ASPD alcohol abusers.

Because patients with ASPD appear to be heterogeneous with respect to treatment response, the question of how to identify in advance those ASPD alcoholics who can benefit from treatment assumes great importance. As mentioned, the ability to experience distress (e.g., anxiety and depression) may predict a positive treatment response in ASPD alcoholics (Alterman and Cacciola 1991). However, a number of clinical features appear to contraindicate psychotherapy in patients with ASPD. These features include a history of violent behavior resulting in serious injury or death, the absence of remorse or the rationalization of antisocial behavior, the inability to develop emotional attachments, and the elicitation of intense fear in the skilled clinician by the patient’s predatory behavior (Gabbard 1990).

Because alcoholics with ASPD can be extremely disruptive to a group treatment program, the clinician should systematically confront the patient’s destructive behavior and ensure that staff or other patients are not harmed or exploited. Common destructive behaviors displayed by these patients include stealing, intimidation, and other forms of aggression. They also may challenge the program structure or otherwise attempt to discredit the program. To minimize such behaviors, the patient should be asked to sign a treatment contract with explicit rules of conduct at admission to the program. This contract should detail the consequences of breaking rules, which should be strictly enforced. Often peer confrontation can be useful in preventing patients from rationalizing drinking and antisocial behavior. In addition, court-ordered treatment may increase the likelihood of successful outcomes.

If, for any reason, the treatment team is unable to enforce the terms of the contract, treatment is likely to fail. This may occur for various reasons, including the absence of an adequately structured environment or a clinician’s fear of being assaulted. Alcoholic patients with moderate-to-severe ASPD may require such highly structured programming that treatment may not be possible in a regular outpatient setting. Under these circumstances, it may be necessary to refer the patient to a long-term residential treatment facility.

Although the role of medications in treating ASPD patients has been limited, certain medications may improve symptoms associated with ASPD, such as aggression or impulsivity. For instance, a 3-month course of lithium was found to be efficacious in managing aggression in prison inmates but had no effect on other antisocial behaviors in this population (Sheard et al. 1976). In addition, some evidence indicates that the SSRI fluoxetine has helped personality-disorder patients with prominent histories of impulsive aggressive behavior (Coccaro and Kavoussi 1997; Coccaro 1998). However, it must be emphasized that the decision to prescribe medications for these patients must be considered carefully. This includes identifying target symptoms that may benefit from medications, considering the potential risks and benefits of a specific medication and avoiding the use of medications with abuse liability.

In the area of personality disorders, dialectical behavior therapy (DBT) has shown efficacy in the treatment of borderline personality disorder (BPD) (Linehan 1993). This disorder is characterized by instability of mood, problematic interpersonal relations, impulsivity, and self-destructive behaviors. Accordingly, DBT focuses on emotion regulation, tolerance of emotional distress, interpersonal effectiveness, and self-management skills. The overlap of symptoms (e.g., impulsive aggression) among ASPD, BPD, and alcohol dependence suggests that DBT might profitably be adapted to address some of the problematic behaviors of heavy drinkers with ASPD.

## Conclusions

The high rates of comorbid psychiatric disorders in patients with alcohol-use disorders require that alcoholic patients undergo careful psychiatric evaluation. However, the diagnosis of a psychiatric disorder in the context of alcohol dependence is complicated by the interactive effects of heavy drinking and psychiatric symptomatology. Similarly, the treatment of alcohol-dependent patients with comorbid disorders is complex, because these patients require help in becoming abstinent from alcohol concomitant with stabilization of their comorbid psychiatric symptoms. Such efforts are important, because untreated psychiatric illness in alcohol-dependent patients is a source of added morbidity and mortality, both directly and by increasing risk for continued heavy drinking. Although additional studies are needed to evaluate the optimal combination of psychotherapy and medication for use in various comorbid subgroups, we have described a number of promising approaches to the treatment of the most common comorbid psychiatric disorders in alcoholics. Despite the difficulties inherent in treating alcoholics with comorbid psychiatric disorders, clinicians can obtain valid diagnoses and deliver efficacious treatment to these patients.

## References

[b1-arh-23-2-144] Alterman AI, Cacciola JS (1991). The antisocial personality disorder diagnosis in substance abusers: Problems and issues. Journal of Nervous and Mental Disease.

[b2-arh-23-2-144] Beck AT, Ward CH, Mendelson M, Mock J, Erbaugh J (1961). An inventory for measuring depression. Archives of General Psychiatry.

[b3-arh-23-2-144] Brown RA, Evans DM, Miller IW, Burgess ES, Mueller TI (1997). Cognitive-behavioral treatment for depression in alcoholism. Journal of Consulting and Clinical Psychology.

[b4-arh-23-2-144] Cacciola JS, Alterman AI, Rutherford MJ, Snider EC (1995). Treatment response of antisocial substance abusers. Journal of Nervous and Mental Disease.

[b5-arh-23-2-144] Ciraulo DA, Jaffe JH (1981). Tricyclic antidepressants in the treatment of depression associated with alcoholism. Journal of Clinical Psychopharmacology.

[b6-arh-23-2-144] Coccaro EM (1998). Clinical outcome of psychopharmacologic treatment of borderline and schizotypal personality disordered subjects. Journal of Clinical Psychiatry.

[b7-arh-23-2-144] Coccaro EM, Kavoussi RJ (1997). Fluoxetine and impulsive aggressive behavior in personality-disordered subjects. Archives of General Psychiatry.

[b8-arh-23-2-144] Cornelius JR, Salloum IM, Ehler JG, Jarrett PJ, Cornelius MD, Perel JM, Thase ME, Black A (1997). Fluoxetine in depressed alcoholics: A double-blind, placebo-controlled trial. Archives of General Psychiatry.

[b9-arh-23-2-144] Donovan SJ, Nunes EV (1998). Treatment of comorbid affective and substance use disorders. American Journal on Addictions.

[b10-arh-23-2-144] Gabbard GO, Gabbard GO (1990). The antisocial patient. Psychodynamic Psychiatry in Clinical Practice.

[b11-arh-23-2-144] Hamilton M (1959). The assessment of anxiety states by rating. British Journal of Medical Psychology.

[b12-arh-23-2-144] Hesselbrock MN, Meyer RE, Keener JJ (1985). Psychopathology in hospitalized alcoholics. Archives of General Psychiatry.

[b13-arh-23-2-144] Hesselbrock VM, Meyer RE, Hesselbrock MN, O’Brien CP, Jaffe JH (1992). Psychopathology and addictive disorders: The specific case of antisocial personality disorder. Addictive States.

[b14-arh-23-2-144] Kessler RC, Mcgonalgle KA, Zhao S, Nelson CB, Hughes M, Eshleman S, Wittchen HU, Kendler KS (1994). Lifetime and 12-month prevalence of DSM-III-R psychiatric disorders in the United States: Results from the National Comorbidity Survey. Archives of General Psychiatry.

[b15-arh-23-2-144] Kessler RC, Crum RM, Warner LA, Nelson CB, Schulenberg J, Anthony JC (1997). Lifetime co-occurrence of DSM-III-R alcohol abuse and dependence with other psychiatric disorders in the National Comorbidity Survey. Archives of General Psychiatry.

[b16-arh-23-2-144] Kranzler HR (1996). Evaluation and treatment of anxiety symptoms and disorders in alcoholics. Journal of Clinical Psychiatry.

[b17-arh-23-2-144] Kranzler HR, Mason B, Modesto-Lowe V, Kranzler HR, Rounsaville B (1998). Prevalence, diagnosis, and treatment of comorbid mood disorders and alcoholism. Dual Diagnosis and Treatment.

[b18-arh-23-2-144] Linehan MM, Onken LS, Blaine JD, Boren JJ (1993). Dialectical behavior therapy for treatment of borderline personality disorder: Implications for the treatment of substance abuse. Behavioral Treatments for Drug Abuse and Dependence.

[b19-arh-23-2-144] Longabaugh R, Rubin A, Malloy P, Beattie M, Clifford PR, Noel N (1994). Drinking outcomes of alcohol abusers diagnosed as antisocial personality disorder. Alcoholism: Clinical and Experimental Research.

[b20-arh-23-2-144] Meyer RE, Meyer RE (1986). How to understand the relationship between psychopathology and addictive disorders: Another example of the chicken and the egg. Psychopathology and the Addictive Disorders.

[b21-arh-23-2-144] Ormrod J, Budd R (1991). A comparison of two treatment interventions aimed at lowering anxiety levels and alcohol consumption amongst alcohol abusers. Drug Alcohol Dependence.

[b22-arh-23-2-144] Powell BJ, Penick EC, Othmer E, Bingham SF, Rice AS (1982). Prevalence of additional psychiatric syndromes among male alcoholics. Journal of Clinical Psychiatry.

[b23-arh-23-2-144] Roy A (1998). Placebo-controlled study of sertraline in depressed recently abstinent alcoholics. Biological Psychiatry.

[b24-arh-23-2-144] Roy-Byrne P, Pages KP, Russo JE, Blume AW, Jaffe C, Kingsley E, Cowley DS, Ries RK A double-blind placebo-controlled trial of nefazodone in the treatment of major depression in alcohol dependent patients. Journal of Clinical Psychopharmacology.

[b25-arh-23-2-144] Sheard MH, Marim JL, Bridges CI, Wagner E (1976). The effect of lithium on impulsive aggressive behavior in man. American Journal of Psychiatry.

